# Effects of different remote ischemia perconditioning methods on cerebral infarct volume and neurological impairment in rats

**DOI:** 10.1038/s41598-023-29475-2

**Published:** 2023-02-07

**Authors:** Shotaro Otsuka, Yuki Itashiki, Akira Tani, Teruki Matsuoka, Seiya Takada, Ryoma Matsuzaki, Kazuki Nakanishi, Kosuke Norimatsu, Yuta Tachibe, Riho Kitazato, Nao Nojima, Shogo Kakimoto, Kiyoshi Kikuchi, Ikuro Maruyama, Harutoshi Sakakima

**Affiliations:** 1 Department of Systems Biology in Thromboregulation, Kagoshima University Graduate School of Medical and Dental Science, 8-35-1 Sakuragaoka, Kagoshima, 890-8520 Japan; 2 Department of Physical Therapy, School of Health Sciences, Faculty of Medicine, Kagoshima University, 8-35-1 Sakuragaoka, Kagoshima, 890-8544 Japan; 3 Division of Brain Science, Department of Physiology, Kurume University School of Medicine, 67 Asahi-machi, Kurume, Fukuoka 830-0011 Japan; 4 Department of Neurosurgery, Kurume University School of Medicine, 67 Asahi-machi, Kurume, Fukuoka 830-0011 Japan

**Keywords:** Neurological disorders, Disease prevention, Therapeutics

## Abstract

Remote ischemic perconditioning (RIPerC) is a novel neuroprotective method against cerebral infarction that has shown efficacy in animal studies but has not been consistently neuroprotective in clinical trials. We focused on the temporal regulation of ischemia–reperfusion by RIPerC to establish an optimal method for RIPerC. Rats were assigned to four groups: 10 min ischemia, 5 min reperfusion; 10 min ischemia, 10 min reperfusion; 5 min ischemia, 10 min reperfusion; and no RIPerC. RIPerC interventions were performed during ischemic stroke, which was induced by a 60-min left middle cerebral artery occlusion. Infarct volume, sensorimotor function, neurological deficits, and cellular expressions of brain-derived neurotrophic factor (BDNF), B-cell lymphoma 2 (Bcl-2), Bcl-2-associated X protein (Bax), and caspase 3 were evaluated 48 h after the induction of ischemia. Terminal deoxynucleotidyl transferase-mediated dUTP-biotin nick-end labeling (TUNEL) was also performed. RIPerC of 10 min ischemia/10 min reperfusion, and 5 min ischemia/10 min reperfusion decreased infarct volume, improved sensorimotor function, decreased Bax, caspase 3, and TUNEL-positive cells, and increased BDNF and Bcl-2 expressions. Our findings suggest RIPerC with a reperfusion time of approximately 10 min exerts its neuroprotective effects via an anti-apoptotic mechanism. This study provides important preliminary data to establish more effective RIPerC interventions.

## Introduction

One in four people in the world will experience a stroke in their lifetime^1^, although it is still difficult to predict the onset of stroke. The time window available for the treatment of stroke is short, so it is important to develop effective neuroprotective measures during the cessation of blood flow to the brain^2^. Recently, a method called remote ischemia conditioning (RIC), in which a pressurized cuff is placed on the upper or lower extremity and repeatedly compressed and released for several minutes to induce protection against ischemia–reperfusion injury, has been studied. RIC is divided into three categories according to the time of intervention: before ischemia (pre-, RIPreC); during ischemia (per-, RIPerC), and after ischemia (post-, RIPostC). Of the three categories, RIPerC is particularly important because it intervenes during the development of cerebral ischemia and may affect patient outcomes.

RIPerC intervention for cerebral infarction has been investigated in human clinical trials as well as animal studies. Although RIPerC was reported to markedly reduce cerebral infarct volume and neurological deficits in animal studies^3–5^, human clinical trials in patients with or suspected of having an acute stroke failed to replicate these beneficial effects^6,7^. Factors contributing to a lack of expected clinical outcomes in RIC were discussed at the 11th Hatter Cardiovascular Workshop, and it was concluded that the establishment of an ideal intervention for RIC is needed^8^. The development of novel effective RIPerC interventions and determining their optimal timepoints might have a positive effect in clinical trials.

RIPerC intervention methods (e.g., pressure point, pressure duration, reperfusion time, and number of cycles) are not standardized, and the most effective methods are unknown. In animal studies, four cycles of 5 min of ischemia and 5 min of reperfusion or three cycles of 10 min of ischemia and 10 min of reperfusion were reported to induce neuroprotective effects^9,10^. However, these results were not reflected in clinical trials^6,7^. To date, no animal studies have focused on how the ischemia and reperfusion times might affect the neuroprotective effects of RIPerC.

Among the many known neurotrophic factors, brain-derived neurotrophic factor (BDNF) is considered the most important factor for neuroprotective effects and recovery from nerve damage. It is essential for neurons because it facilitates their survival and development, and modulates synaptic plasticity, memory, and neuroprotection^11^. Many studies have reported an association between BDNF expression and acute cerebral infarction. For example, lower serum BDNF levels were associated with increased infarct volume in acute stroke^12^. Furthermore, high BDNF levels in acute stroke were associated with improved neurological outcomes^13^. Other studies of brain tissues from patients with acute stroke reported that decreased B-cell lymphoma-2 (Bcl-2) gene expression and protein levels increased the expression of Bcl-2-associated X protein (Bax; a member of the Bcl-2 protein family), which significantly increased apoptosis and exacerbated neurological dysfunction^14–16^. Therefore, the inhibition of cellular apoptosis within the cerebral infarction penumbra may protect nerve function.

The aim of this study was to examine RIPerC interventions with a focus on setting optimal ischemia–reperfusion times in a rat model of ischemic stroke and to collect preliminary data that can be applied to future clinical studies. Specifically, we focused on examining the optimal ischemia and reperfusion times in RIPerC to determine how different ischemia and reperfusion times in RIPerC affected the cerebral infarct volume, and to explore effective intervention methods. Furthermore, we focused on the neuronal apoptosis pathway by examining BDNF, Bcl-2, Bax, Bax/Bcl-2 ratio, and caspase 3 to determine whether adjusting the ischemia–reperfusion time of RIPerC altered the expression kinetics of these factors and affected the neuroprotective effects of RIPerC.

## Results

### RIPerC reduces infarct volume and sensorimotor deficits after focal cerebral ischemia (depending on intervention time)

**Figure 1:**
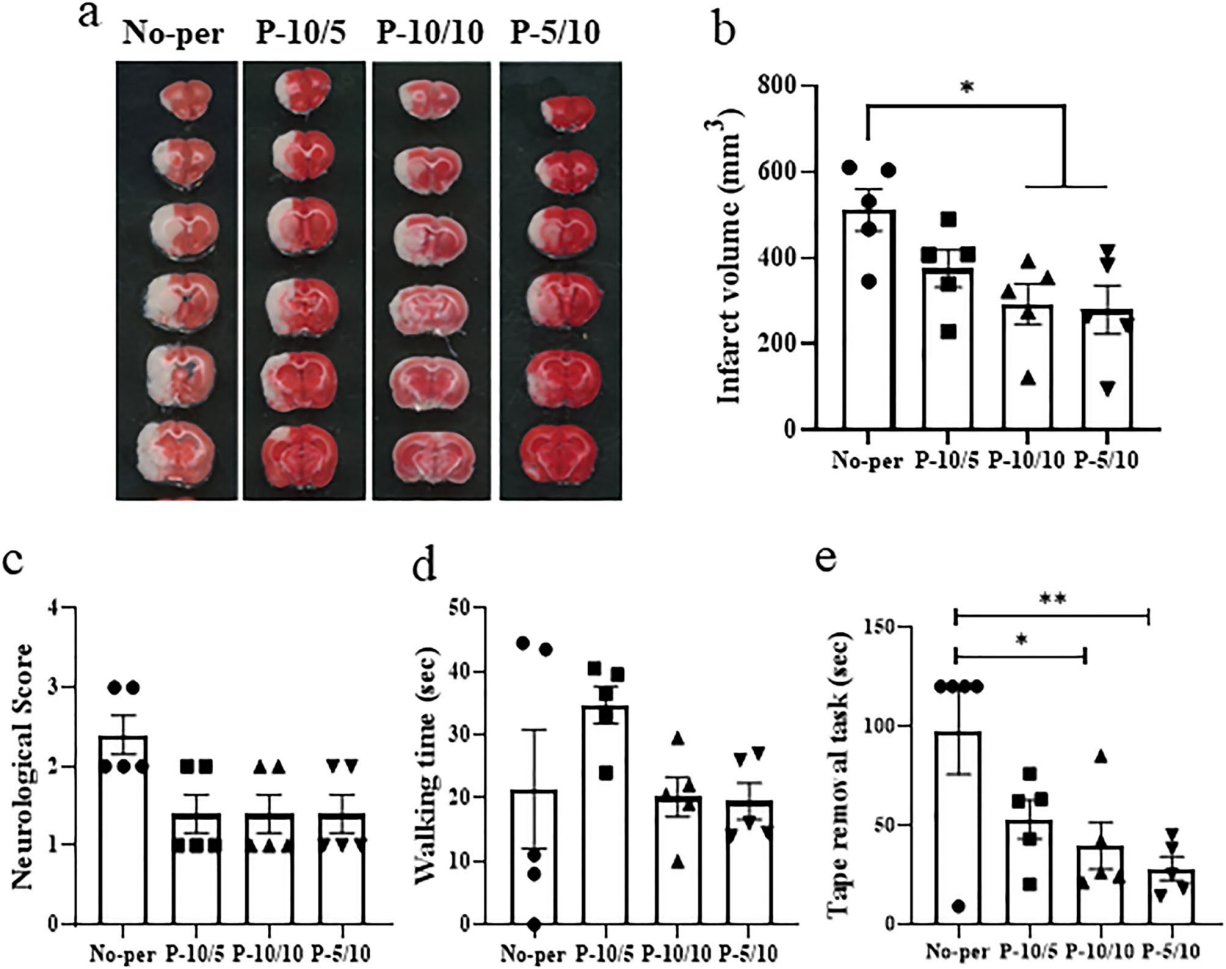
Neuroprotective effects of different intervention times of RIPerC after MCAO. Representative TTC-stained cerebral sections of the No-per, P-10/5, P-10/10, and P-10/5 groups (**a**). The infarct volume in the P-10/10 and P-5/10 groups was significantly decreased compared with the No-per group. The P-10/5 group had no reduction in infarct volume compared with the No-per group (**b**). The motor cortex of the ischemic penumbra surrounding the necrotic lesions (*) of the rectangle areas (A; the third of seven consecutive TTC sections from the cranial to the caudate region, containing the motor cortex) were used for the quantitative analysis of immunolabeled areas. The neurological scores (**c**), sticky-tape removal task times (**d**), and rotarod balancing times (**e**) are shown. Neurological scores were significantly reduced in all three groups compared with the No-Per group. The sticky-tape removal task had significantly shorter times in the P-10/10 and P-10/5 groups than in the No-per group. There were no significant differences in rotarod results among all groups. Data are presented as the mean ± SE (*n* = 5). *p < 0.05.

We investigated the effects of the duration of ischemia and reperfusion in RIPerC on infarct volume. The RIPerC method of four cycles of 5-min ischemia/5-min reperfusion, reported in a previous study, did not reduce infarct volume in the present study (Supplementary Fig. 1). Therefore, we adjusted the ischemia and reperfusion times to perform three sets of 10-min ischemia/10-min reperfusion (P-10/10), which has also been reported previously^9^. There was a significant reduction in cerebral infarct volume in the P-10/10 group compared with the no RIPerC (No-per) group (Fig. 1a,b). The effects of ischemia and reperfusion length on cerebral infarct volume were then evaluated using three cycles of 10-min ischemia/5-min reperfusion (P-10/5), and 5-min ischemia/10-min reperfusion (P-5/10), respectively. Compared with the No-per group, the P-10/5 group showed no reduction in infarct volume, whereas the P-5/10 group had a significant reduction (Fig. 1a,b). These findings indicate that the neuroprotective effect of RIPerC is attenuated by shortening the reperfusion time.

We then evaluated whether RIPerC affected neurological damage or sensorimotor function after stroke. Regardless of the RIPerC intervention method, neurological deficits were mild, but not significantly different from the No-per group (Fig. 1c). Similarly, there were no significant differences between the groups in the rotarod test, which assesses balance function and endurance (Fig. 1d). In the sticky-tape removal test, which evaluates sensorimotor function, reaction times were significantly shorter in the P-10/10 and P-5/10 groups compared with the No-per group (Fig. 1e).

### RIPerC decreases neuronal cell death by increasing BDNF and Bcl-2 and suppressing Bax and caspase 3

**Figure 2:**
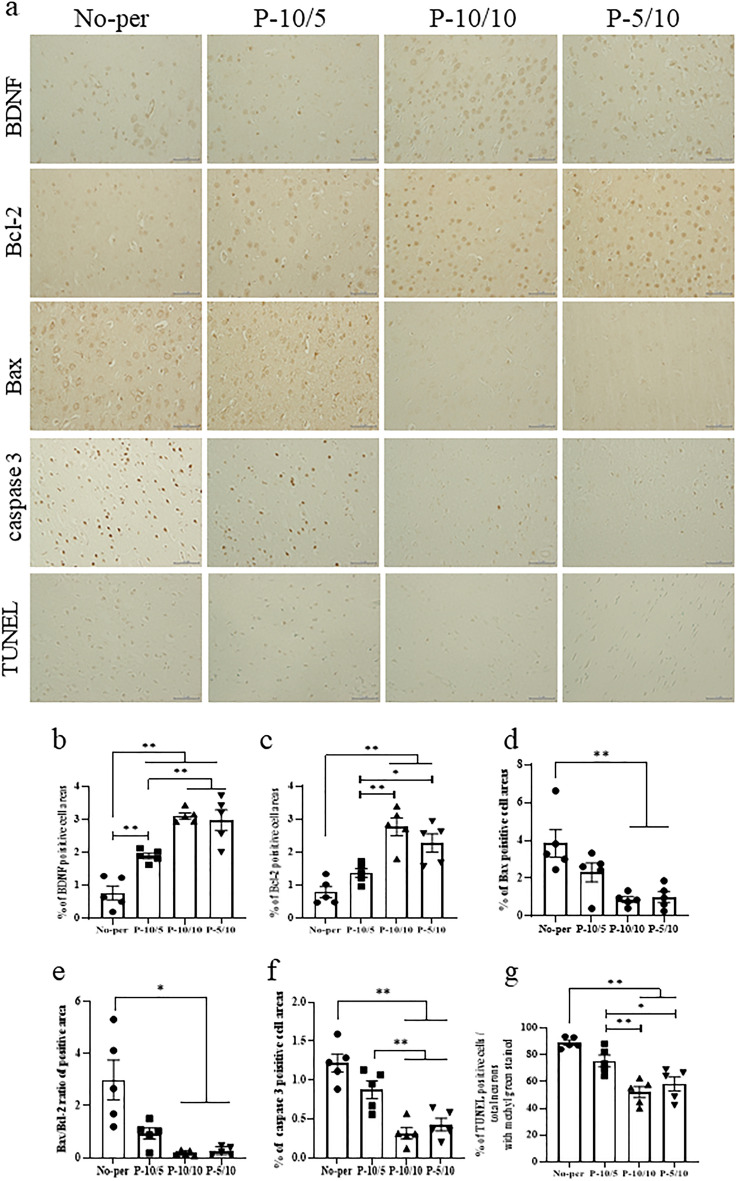
BDNF, Bcl-2, Bax, Bax/Bcl-2 ratio, and caspase 3 expressions and TUNEL staining in the ischemic penumbra after MCAO. Photomicrographs of BDNF, Bcl-2, Bax, caspase 3, and TUNEL staining (**a**); graphs of the areas of BDNF- (**b**), Bcl-2- (**c**), Bax- (**d**), Bax/Bcl-2 ratio- (**e**), caspase 3- (**f**), and TUNEL-positive (**g**) cells. BDNF expression was significantly increased in the P-10/5, P-10/10, and P-5/10 groups compared with the No-per group. The P-10/10 and P-5/10 groups had significantly increased BDNF expression compared with the P-10/5 group. Bcl-2 expression was significantly increased in the P-10/10 and P-10/5 groups compared with the No-per group. The P-10/10 and P-5/10 groups had significantly increased Bcl-2 expression compared with the P-10/5 group. Bax and caspase 3 expressions were significantly decreased in the P-10/10 and P-10/5 groups compared with the No-per group. The Bax/Bcl-2 ratio increased after MCAO, but the P-10/10 and P-5/10 groups had significantly decreased Bax/Bcl-2 ratios compared with the No-per group. The P-10/10 and P-5/10 groups exhibited significantly decreased caspase 3 expression compared with the P-10/5 group. The P-10/10 and P-10/5 groups had a significantly reduced number of TUNEL-positive cells surrounding the lesion compared with the P-5/10 and No-per groups. Data are presented as the mean ± SE (*n* = 5). *p < 0.05, **p < 0.01; scale bar in all panels = 50 μm.

We investigated whether RIPerC affected the expression of BDNF, a factor that induces neuroprotective effects. BDNF immunoreactivity was significantly increased in sensorimotor areas surrounding the necrotic lesions in the three groups with RIPerC after middle cerebral artery occlusion (MCAO) (Fig. 2a,b). Among the RIPerC groups, BDNF expression was significantly increased in the P-10/10 and P-5/10 groups compared with the P-10/5 group (Fig. 2a,b). Bcl-2 immunoreactivity was also increased in the peri-necrotic lesion areas of the P-10/10 and P-5/10 groups compared with the No-per and P-10/5 groups (Fig. 2a,c). Furthermore, Bax immunoreactivity was reduced in the regions surrounding the necrotic lesion in the P-10/10 and P-5/10 groups compared with the No-per group (Fig. 2a,d). The Bax/Bcl-2 ratio was reduced by the RIPerC intervention, with significant reductions in the P-10/10 and P-5/10 groups compared with the No-per group (Fig. 2a,e). We then examined the expression of caspase 3, the final determinant of apoptosis, after RIPerC. Caspase 3 immunoreactivity was decreased in the peri-necrotic lesion areas of the P-10/10 and P-5/10 groups compared with the No-per and P-10/5 groups (Fig. 2a,f). Moreover, TUNEL staining in the peri-necrotic lesion areas revealed that neuronal death was reduced in the P-10/10 and P-5/10 groups compared with the No-per and P-10/5 groups (Fig. 2a,g).

**Figure 3:**
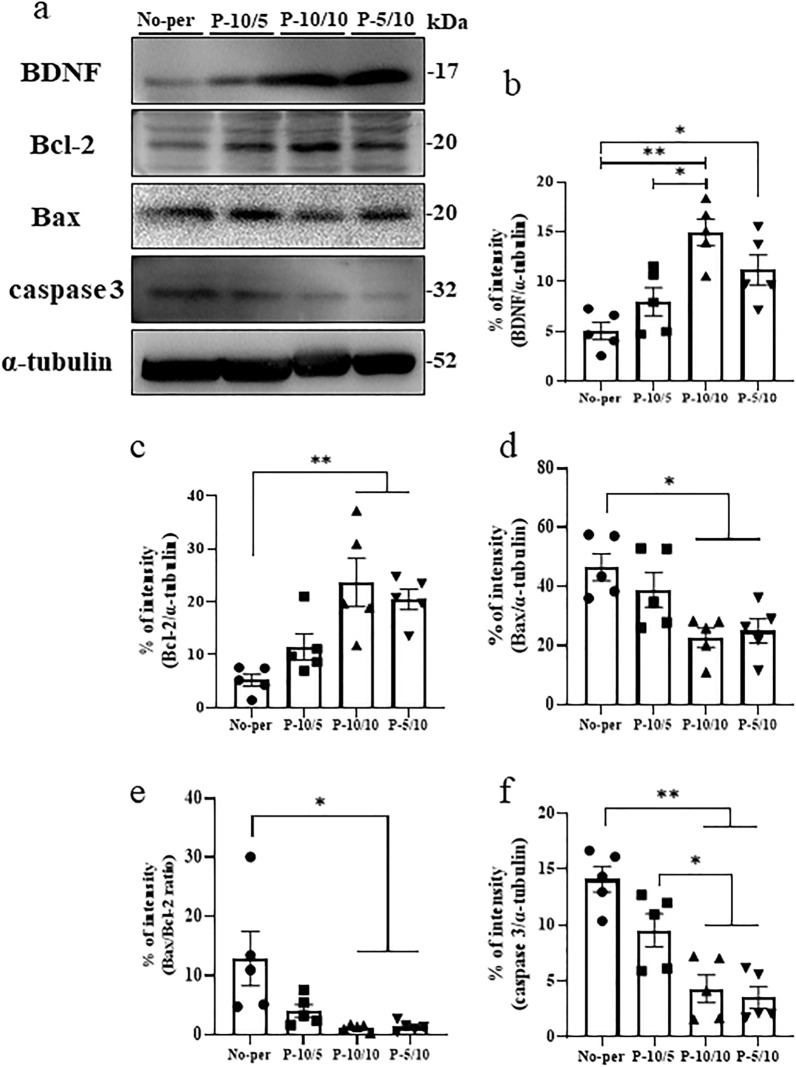
Effects of RIPerC on neuronal apoptosis and the BDNF/Bcl-2/Bax/caspase 3 pathway after MCAO. Representative western blots and semi-quantitative analyses of the expressions of BDNF, Bcl-2, Bax, Bax/Bcl-2 ratio, and caspase 3 in the ipsilateral hemisphere (**a**). BDNF expression was significantly increased in the P-10/10 and P-10/5 groups compared with the No-per group. The P-10/10 group had increased BDNF expression compared with the P-10/5 group (**b**). Bcl-2 expression was significantly increased in the P-10/10 and P-10/5 groups compared with the No-per group (**c**). Bax expression was significantly decreased in the P-10/10 and P-5/10 groups compared with the No-per group (**d**). The Bax/Bcl-2 ratio was significantly reduced in the P-10/10 and P-5/10 groups compared with the No-per group, although there was no significant reduction in the P-10/5 group compared with the No-per group (**e**). The P-10/10 and P-5/10 groups had decreased caspase 3 expression compared with the P-10/5 and No-per groups (**f**). The band in (**a**) is the cut out target band only, and the membrane before cutting is shown in the supplemental data. Data are presented as the mean ± SE (*n* = 5). *p < 0.05, **p < 0.01.

Western blot analysis also demonstrated that protein levels of BDNF in the ipsilateral hemisphere were significantly increased in the P-10/10 and P-5/10 groups compared with the No-per group (Fig. 3a,b). In contrast, there was no significant difference in BDNF protein expression between the P-10/5 and No-per groups (Fig. 3a,b). The protein levels of Bcl-2 in the ipsilateral hemisphere were significantly increased in the P-10/10 and P-5/10 groups compared with the No-per group (Fig. 3a,c). Additionally, Bax protein levels were significantly decreased in the P-10/10 and P-5/10 groups compared with the No-per group (Fig. 3a,d). Furthermore, the Bax/BCl-2 ratio was increased in the No-per group and decreased after RIPerC, whereas the Bax/Bcl-2 ratio was decreased significantly in the P-10/10 and P-5/10 groups compared with the No-per group (Fig. 3a,e). Moreover, caspase 3 protein levels were decreased in the P-10/10 and P-5/10 groups compared with the No-per and P-10/5 groups (Fig. 3a,f). There was no significant difference in caspase 3 protein levels in the ipsilateral hemisphere between the P-10/5 and No-per groups (Fig. 3a,f).

## Discussion

In the current study, we investigated the effects of different RIPerC intervention methods—with different ischemia and reperfusion times—on infarct volume. We found that different RIPerC intervention methods affected the observed neuroprotective effects. Shorter reperfusion times were found to attenuate the observed infarct volume reduction effect of RIPerC. Furthermore, our results indicated that the neuroprotective mechanism of RIPerC involved increased BDNF expression and inhibition of the Bcl-2/Bax/caspase 3 apoptotic pathway.

We hypothesized that the uncertain intervention methods of RIPerC have reduced its effectiveness in clinical trials^6,7^, and that establishing more effective RIPerC intervention methods in animal studies might demonstrate its effectiveness in human clinical trials. The present study investigated the effects of different lengths of ischemia and reperfusion time on the neuroprotective effects of RIPerC against ischemic stroke. The effect of RIPerC on reducing cerebral infarct volume was decreased with a shorter reperfusion time. These results suggest that determining the optimal reperfusion time is important for the neuroprotective effects of RIPerC. Similarly, previous studies of RIPostC have determined the most effective combination of RIC cycle numbers and ischemia/reperfusion times. A previous study confirmed that two cycles of 15-min ischemia/15-min reperfusion significantly promoted the reduction of cerebral infarct volume and improvement of neurological deficits after stroke, indicating an ischemia time of 10–15 min is necessary^17,18^. Furthermore, Ma et al. reported that three cycles of RIPerC with 15 min of ischemia and 15 min of reperfusion reduced the cerebral infarct volume^19^. Interestingly, the present study showed that three cycles of ischemia time as short as 5 min and 10 min of reperfusion reduced the cerebral infarct volume in contrast to the reported 10–15 min of ischemia. Furthermore, preconditioning (PC)—in which ischemia is induced by the direct compression of blood vessels—had no effects on infarct volume when an ischemic stroke occurred the day after a 15-min PC intervention; however, a reperfusion period of at least 3 days after PC reduced the infarct volume. That is, a reperfusion period of at least 3 days after PC is necessary for the induction of ischemic tolerance with strong neuroprotective effects^20^. Thus, the effect of RIPerC may be altered by prolonging the reperfusion time even when the ischemia time is short. Whether the ischemia time or reperfusion time is more important is not known, but further validation is needed to determine whether an ischemia time of 5 min is the minimum necessary to obtain a neuroprotective effect.

It remains unclear whether RIPerC increases the expression of BDNF, which is important for neuronal protection during acute cerebral infarction. BDNF is widely distributed throughout the brain and plays an important role in the neural repair process^21^. Several studies have demonstrated that RIPostC after cerebral ischemia protected against degenerative events in rats via increased BDNF expression^22^. Additionally, we previously reported that increased BDNF expression was involved in the neuroprotective effects of preventive exercise, with an observed reduction in stroke volume as well as motor-sensory dysfunction^23^. In the present study, RIPerC increased BDNF expression in all groups compared with the No-Per group. According to previous studies^22,23^, BDNF expression was increased by RIPreC, and BDNF mRNA and protein expressions were increased by RIPostC. Taken together, these findings suggest that ischemia–reperfusion of the limbs increases BDNF expression in the brain regardless of whether it occurs during the pre-, per-, or post-period. Notably, in the present study, the group with a reperfusion time of 5 min had a smaller increase in BDNF expression than the group with a reperfusion time of 10 min. Similarly, a shorter reperfusion time resulted in a smaller increase in BDNF-positive cells in the penumbra region. Although it is difficult to clearly explain this phenomenon using the present findings, the reperfusion time is very likely to affect BDNF expression in RIPerC interventions.

We also investigated the expression of Bcl-2, an anti-apoptotic factor that—like BDNF—is important for recovery from acute stroke. Bcl-2 is highly expressed in the embryonic and developing mammalian brain^24^. The regulation of apoptosis after brain injury by altering the levels of anti-apoptotic molecules such as Bcl-2 is well known. For example, the administration of an inhibitor to the brain to decrease endogenous Bcl-2 exacerbated neuronal death after ischemia^25,26^. Moreover, in an intervention study of RIPerC in 30 patients with rheumatic valve disease who underwent double valve (mitral and aortic valve) replacement surgery, RIPerC had a protective effect against ischemia–reperfusion injury on the myocardium, and Bcl-2 expression in harvested atrial myocardial tissue was increased, indicating that apoptosis of the atrial myocardium was inhibited^27^. Furthermore, previous studies of RIPreC revealed that increased Bcl-2 expression was involved in the effects of RIPerC (four cycles of 5-min ischemia/5-min reperfusion) on reducing cerebral infarct volume^28^. Thus, our results are consistent with those of previous studies and indicate that Bcl-2 expression in the brain is increased by RIPerC.

In the rat brain, the expressions of various apoptotic proteins are upregulated in the penumbra during the first few hours after cerebral infarction. For example, in the rat cerebral cortex, the overexpression of caspase-1, -3, -6, -8, and -9 was observed in the infarct nucleus as early as 0.5–1 h after MCAO, and this overexpression was involved in the expansion of the infarct volume^29–31^. Caspases are major factors in neuronal apoptosis related to their ability to inhibit caspase 3, which is the final determinant of apoptosis. In the current study, we therefore observed the effects of RIPerC on caspase 3. RIPerC suppressed caspase 3 expression, and longer reperfusion times had a more significant effect. Notably, BDNF protected neurons by suppressing caspase 3^32^. Moreover, our previous study demonstrated that the neuroprotective effects of preconditioning exercise before stroke were associated with increased BDNF expression and that the inhibition of caspase 3 by BDNF was a factor in reducing the infarct volume^23^.

The Bax protein, a member of the Bcl-2 family, promotes apoptosis^33,34^. Bcl-2 interacts with Bax and inhibits Bax-induced apoptosis. A previous study of preconditioning exercise also reported a decrease in cerebral infarct volume as the result of a decrease in the Bax/Bcl-2 ratio and the inhibition of caspase 3 activation after cerebral infarction^35^. Similar to this previous study, the present results showed that Bcl-2 expression was increased and Bax expression was decreased after reperfusion with RIPerC for 10 min, suggesting that caspase 3 activation was reduced and neuronal apoptosis was suppressed. When considering mechanisms other than the inhibition of apoptosis, RIPerC was also reported to modulate immune responses and suppress the expression of inflammatory cytokines, which may also promote its neuroprotective effects during acute cerebral infarction^28^. Additionally, the increased expression of BDNF by RIPerC and suppression of the Bcl-2/Bax/caspase 3 pathway also reduced TUNEL-positive cell numbers, which together might have suppressed neuronal cell death and reduced cerebral infarct volume.

One limitation of this study is that we have not been able to examine the mechanisms of action that underlies our results. Analysis of brain tissue alone is limited for elucidating the mechanism of neuroprotective effects of different ischemia/reperfusion times. A comprehensive analysis needs to include a variety of tissues, such as skeletal muscle, vascular endothelium, and blood. In this study we only collected brain tissue samples because we focused on the apoptotic pathway after cerebral infarction as the neuroprotective effect of RIPerC. Therefore, we did not elucidate the mechanism in more detail. However, we consider that in future studies the mechanism can be determined by analyzing each tissue. In addition, a previous RIPC study using human neurons showed that RIPC plasma exerts an anti-apoptotic effect on human HB1.F3 neural stem cells under oxygen glucose deprivation, which mimics cerebral ischemia^36^. The effectiveness of the intervention method used in this study should be verified using in vitro studies of human neural cells, as previously described^36^.

Although we were able to demonstrate the importance of reperfusion time, we do not know how prolonged reperfusion might enhance the neuroprotective effects. Pulse monitoring to assess the effects of blood flow may help to clarify this point. Although cerebral infarct volume was influenced by reperfusion time, neurological scores were not significantly altered. This finding suggests that the intervention conditions set in the present study may not be optimal; a reperfusion time of 10 min or longer may elicit greater effects. Additionally, the effect of ischemia duration on neuronal apoptotic pathways needs to be investigated, which might be clarified by conducting intervention experiments in settings with ischemia durations of 5 min or less and 10 min or more. It is possible that no significant difference in neurological deficits was observed in the current study because of the relatively short duration of the experiment, and a significant improvement in neurological deficits may be observed if longer-term experiments are conducted. Furthermore, although the mechanism of RIPerC in this study appeared to be via the inhibition of neuronal apoptosis, oxidative stress and inflammatory responses are also involved in infarct volume and should be examined in the future. Finally, as well as ischemia–reperfusion time, the number of cycles, site of ischemia application, and number of points of pressure application in RIPerC must also be considered. Once these important aspects are clarified, the marked effects of RIPerC that have been observed in animal studies might be applicable to clinical practice.

In the present study, we examined the effects of RIPerC intervention methods—focusing on ischemia and reperfusion time—on infarct volume. The P-10/10 and P-5/10 RIPerC conditions significantly decreased the infarct volume, improved sensorimotor function, decreased Bax, Bax/Bcl-2 ratio, caspase 3, and TUNEL-positive cells, and increased BDNF and Bcl-2 expressions. Our findings suggest that RIPerC with a reperfusion time of approximately 10 min exerts neuroprotection via the anti-apoptotic effects of the neuroprotective factor BDNF, the mitochondria-mediated apoptotic pathway, and the inhibition of caspase 3 through increased Bcl-2 and decreased Bax and Bax/Bcl-2 ratio. The results of the current study provide important preliminary data about how to increase the effectiveness of RIPerC interventions and will encourage future research into RIPerC intervention methods.

## Methods

### Animals

All experimental procedures performed were in accordance with the Reporting of In Vivo Experiments (ARRIVE) guidelines and all experiment plans were approved by the Ethics Committee of the Kagoshima University Institute for Experimental Animal Science (Approval No.: MD21088). Twenty-eight male Sprague Dawley rats (CLEA Japan, Inc., Shizuoka, Japan) (7 weeks old, weight 240–270 g) were used in this study. The number of animals used was determined with reference to previous studies^9,37^. The rats were housed in the same manner as in our previous experiments with rats^23,38^. An effort was taken to reduce the number of animals used. The rats were housed in pairs in a temperature-controlled environment (22 ± 1 °C) with a 12-h light/dark cycle, and food and water were available ad libitum. Rats were caged for 1 week after giving birth for pre-experimental acclimation.

### RIPerC protocol

**Figure 4:**
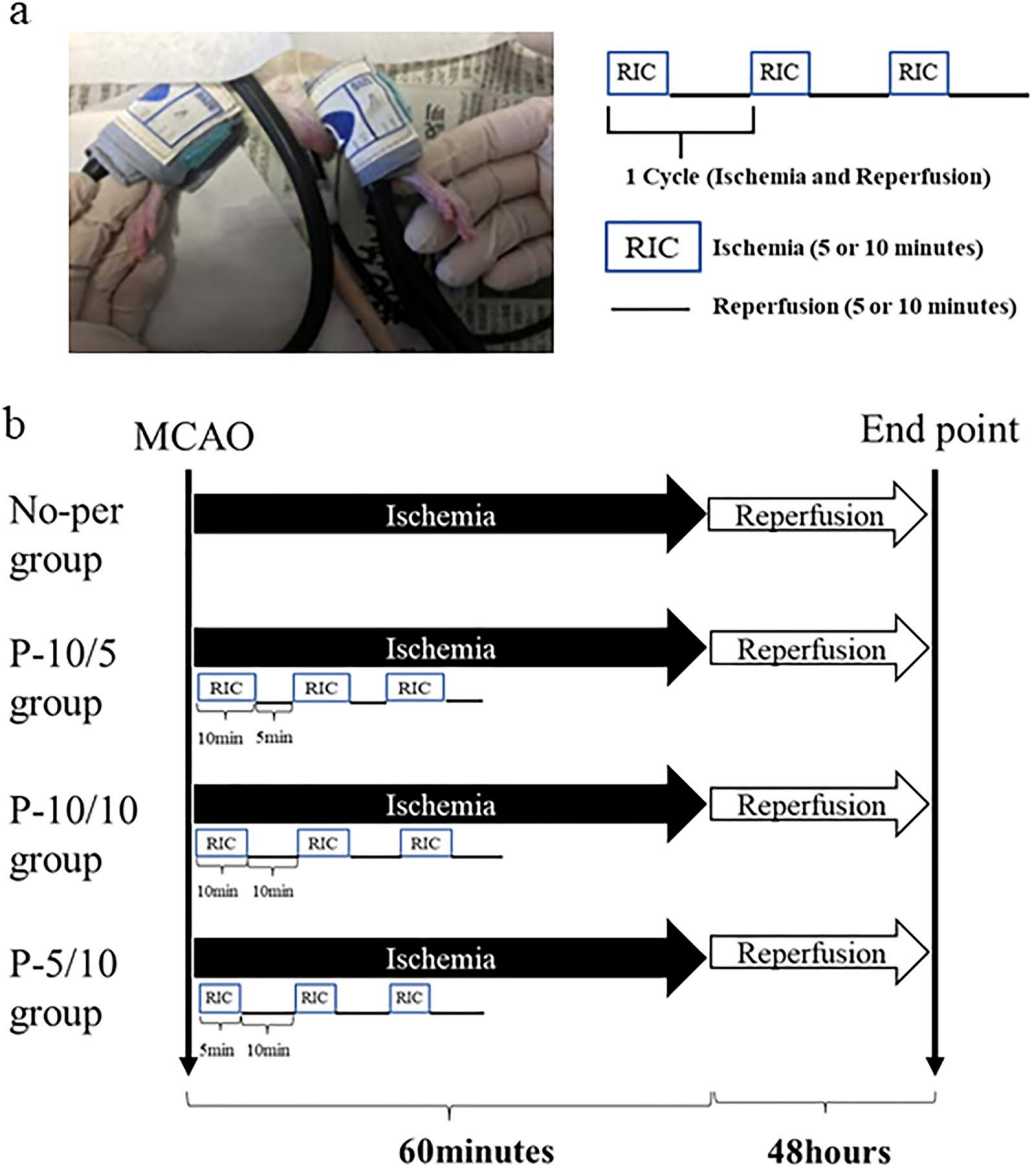
Experimental protocol; schematic diagram of remote ischemic perconditioning (RIPerC) groupings and interventions. RIPerC was performed in anesthetized rats with cuffs applied to pressurize both thighs (**a**). The RIPerC intervention was divided into three groups by adjusting the ischemia–reperfusion times (**b**). One intervention group consisted of three cycles of one set of 10 min of ischemia and 5 min of reperfusion (P-10/5 group, n = 5), the second group consisted of three cycles of one set of 10 min of ischemia and 10 min of reperfusion (P-10/10 group, n = 5), and the third group consisted of three cycles of one set of 5 min of ischemia and 10 min of reperfusion (P-5/10 group, n = 5). The group without RIPerC wore a cuff that was not pressurized during ischemia (No-per group, n = 5).

The experimental protocol is illustrated in Fig. 4. The intervention was based on previous studies by Lv et al.^4^ Three RIPerC intervention protocols were performed to examine the optimal ischemia and reperfusion times. For RIPerC, elastic pressure cuffs (pediatric sphygmomanometers) were applied to both hindlimb thighs, and one of three patterns of intervention was then performed during cerebral ischemia. The applied pressure was 180–200 mmHg and cyanosis appeared in the toes of the rats, confirming ischemia; blocking blood flow in the femoral artery in mice has been reported to cause cyanosis, swelling of the extremities, loss of dorsal pulses in the legs, and a decrease in skin temperature^4^.

The rats were divided into one of four intervention groups. One intervention consisted of three cycles of one set of 10 min of ischemia and 5 min of reperfusion (P-10/5 group, n = 5); the second intervention consisted of three cycles of one set of 10 min of ischemia and 10 min of reperfusion (P-10/10 group, n = 5); and the third intervention consisted of three cycles of one set of 5 min of ischemia and 10 min of reperfusion (P-5/10 group, n = 5). The fourth group did not undergo RIPerC and wore a cuff that was not pressurized during ischemia (No-per group, n = 5). The experiment was performed only once with this protocol (Fig. 4).

### Induction of MCAO in rats

The MCAO model was created as previously reported^23,38^. Medetomidine 0.3 mg/kg, midazolam 2.0 mg/kg, and butorphanol 2.5 mg/kg were administered as intraperitoneal anesthesia to rats. The intraoperative rectal temperature was maintained at 37.0 ± 0.5 °C using a heated blanket (KN-474, NATSUME, Tokyo, Japan) via a temperature control system with feedback control. A stroke was triggered by left MCAO for 60 min using intraluminal filaments as previously described^23,39^. The filaments were then pulled out to establish reperfusion.

### Neurological score assessment

A neurological assessment was made on a five-point scale from zero to four, as in our previous studies^23,39^. The following scales were used: 0 = no apparent disability, 1 = right forelimb flexion, 2 = decreased gripping strength of right forelimb when tail is pulled, 3 = spontaneous movements in all directions during right turn only when tail is pulled, and 4 = spontaneous movements during right turn.

### Motor function assessment

The motor function and balance of rats were tested by a rotarod task (MK-670, Muromachi Kikai Co., Ltd., Tokyo, Japan) as in our previous studies^23,35^. The rotational speed was increased from 0 to 25 rpm in increments of 2.5 rpm every 6 s. The longest time (in seconds) to fall was used for evaluation. Three trials were performed for each animal.

### Sensorimotor function evaluation

Sensorimotor functions were evaluated using the sticky-tape removal test, as in our previous studies^38,40^. Square sticky-tape labels (169 mm^2^) that covered the palmar surface of each forepaw were used as bilateral tactile stimuli. Time spent peeling the labels from the forelimbs was scored in two trials for each forelimb. The analysis used the shorter of the two time periods. The allowable maximum time to peel off the label was 120 s.

### Euthanasia

Euthanasia was performed based on our previous study^40^. At the completion of the trial, the rats were deeply anesthetized with intraperitoneal injections of sodium pentobarbital (100 mg/kg). Then, they were transcardially perfused with saline, decapitated, and tissues were sampled.

### Evaluation of ischemic infarct

The method of measuring infarct volume was based on that of our previous studies^23,38^. Brains were extracted and sliced into seven coronal sections 2 mm thick from the frontal lobe apex using a brain slicer (MK-RC-01, Muromachi Machinery Co., Ltd, Tokyo, Japan). Thereafter, the sections were stained by immersion in 1% 2,3,5-triphenyltetrazorlium chloride (TTC) solution in phosphate buffered saline (PBS, pH 7.4) for 15 min at 37 °C. After staining, the sections were scanned and the ischemic infarct volume was measured in each group (n = 5) using image processing software (ImageJ 1.46r; National Institutes of Health, Bethesda, MD, USA). The total volume of the infarct area (mm^2^) was multiplied by the thickness of the brain section to obtain the infarct volume. Quantitative analysis of infarct volume was performed by two people blinded to the grouping of animals, and the analysis was performed on the mean infarct volume of each animal.

### Histology and immunohistochemistry

Immunohistochemical staining was carried out according to our previous studies^38,40^. After ischemic stroke, brain sections were stained with hematoxylin–eosin (H.E.) stain and evaluated histologically. Furthermore, coronal brain slices were stained using the following primary antibodies: rabbit anti-BDNF (ab108319; Abcam PLC, Waltham, MA, USA, 1:100); rabbit anti-Bcl-2 (bs-4563R; Bioss Inc., Woburn, MA, USA, 1:200); rabbit anti-Bax (ab32503; Abcam PLC, 1:200); and rabbit anti-caspase 3 (196771-AP; Proteintech Group, Inc., Rosemont, IL, USA, 1:500). These were then deparaffinized, rehydrated, and blocked for 15 min with methanol containing 0.9% hydrogen peroxide for endogenous peroxidase. Then, the sections were washed three times for 5 min each with PBS (pH 7.6) and blocked with 10% skim milk in PBS for 20 min. The sections were then incubated with each of the aforementioned primary antibodies overnight at 4 °C, washed three times for 10 min each in PBS, incubated with peroxidase-conjugated dextran polymer (EnVision; Agilent Dako, Santa Clara, CA, USA) and goat anti-rabbit IgG, and incubated at 25 °C for 60 min. After washing the sections with PBS, immunoreaction was visualized by 3,3′-diaminobenzidine staining.

### Quantitative analysis of immunolabeled areas

Quantitative analysis of immunolabeled areas was performed based on our previous studies^23,38^. In coronal sections, areas containing cells positive for BDNF, Bcl-2, Bax, and caspase-3 were quantitatively measured (the third of seven consecutive sections from the cranial to caudate regions; motor cortex area; Supplementary Fig. 2) using imaging software (ImageJ 1.46r). The two regions of the motor field of each immunostained section (each region was 0.36 mm^2^) were photographed at 20 × magnification using a microscope with a camera. The average ratio of each immunolabeled area in the two regions of the motor cortex (immunolabeled area/total area) was then calculated. Quantitative analysis was performed for each immunolabeled region by two persons blinded to the grouping. Immunohistochemical staining and quantitative analysis of immunolabeled areas were performed twice to confirm the reliability of the results (Supplementary Fig. 3).

### Terminal deoxynucleotidyl transferase dUTP nick-end labeling (TUNEL) assay

The TUNEL assay was carried out in the ischemic penumbra region using our previously reported methods^35,40^. The procedure was performed twice to confirm the reliability of the results (Supplementary Fig. 3). We used an apoptosis in situ detection kit (Wako Pure Chemicals, Osaka, Japan) according to the manufacturer’s protocol, and sections were counterstained with methyl green. We recognized TUNEL-positive cells morphologically as apoptotic neurons and the number of TUNEL-positive neurons was counted in coronal sections. The two cortical areas ipsilateral to the vascular puncture site were imaged using a microscope and camera with a magnification of 20 × so that the fields of view did not overlap (Supplementary Fig. 2). Quantitative analysis of TUNEL-stained neurons was performed by two individuals blinded to the grouping and analyzed using the mean value. The average number of TUNEL-positive neurons in the groups was then determined.

### Western blotting

Western blotting and semi-quantitative analysis methods were performed as previously described^37,40^. The procedures were performed twice to confirm the reliability of the results (Supplementary Figs. 4–6). Western blotting was performed to detect the protein expressions of BDNF, Bcl-2, Bax, and caspase 3 in the ipsilateral hemisphere of No-per, P-10/5, P-10/10, and P-5/10 rats (n = 5 per group) using the following antibodies: rabbit anti-BDNF (ab108319; Abcam PLC, 1:500); rabbit anti-Bcl-2 (bs-4563R; Bioss Inc, 1:500); rabbit anti-Bax (ab32503; Abcam PLC, 1:1000); rabbit anti-caspase 3 (196771-AP; Proteintech Group, Inc, 1:1000); and mouse anti-α-tubulin (66031-1 g; Proteintech Group, Inc, 1:2000). Briefly, cortical tissues were dissected on ice and homogenized in tissue protein extraction reagent (Thermo Scientific/Pierce T-PER, 78,510; Thermo Fisher Scientific, Waltham, MA, USA). From each sample, approximately 10 μg of protein was loaded onto a 4–15% Mini-PROTEAN Precast Gel (Bio-Rad Laboratories, Inc., Hercules, CA, USA) and then transferred to a nitrocellulose membrane. After a 1-h block at 25 °C in Tris-buffered saline/Tween-20 buffer (0.01 M Tris–HCl, pH 7.5, 0.15 M NaCl, 0.05% Tween-20) containing 5% skim milk, the membrane was incubated with the primary antibody at 4 °C overnight and incubated with horseradish peroxidase-conjugated secondary antibody at a temperature of 25 °C for 1 h. For the secondary antibodies, goat anti-rabbit IgG H&L (HRP) (ab6721; Abcam PLC, 1:1000) and goat anti-mouse IgG H&L (HRP) (ab97023; Abcam PLC, 1:1000) were used. Protein bands were visualized using a chemiluminescence device (WSE-6100 LuminoGraph I; ATTO Corp., Tokyo, Japan) and measured using image processing software (ImageJ 1.46r). Protein levels were measured semiquantitatively using α-tubulin as an internal loading control.

### Statistical analyses

All statistical analyses were performed using the Shapiro–Wilk test followed by parametric and nonparametric tests. For the analysis of neurological scores, the Kruskal–Wallis test was used. Outcomes of the adhesive tape removal and rotor rod tests, expressions of BDNF, Bcl-2, Bax, and caspase-3, and the number of TUNEL-positive cells were analyzed using one-way analysis of variance (ANOVA) and Tukey’s post hoc test for multiple comparisons. A p value < 0.05 was considered statistically significant. The data were expressed as the mean ± standard error (SE). All data were analyzed using IBM SPSS Statistics for Windows, version 26 (IBM Corp., Armonk, NY, USA).

### Ethical approval

All experimental procedures complied with the Animal Research: Reporting of In Vivo Experiments (ARRIVE) guidelines and the experimental protocol was approved by the Ethics Board of the Institute of Laboratory Animal Sciences of Kagoshima University (approval number: MD21088). Additionally, the International Council for Laboratory Animal Science (ICLAS) Ethical Guideline for Researchers, and American Veterinary Medical Association (AVMA) guidelines were followed with regard to pain relief and euthanasia for animals.

## Supplementary Information

Supplementary Information.

## Data Availability

The data and raw materials presented in this report are available from the corresponding authors upon request.

## References

[1] Feigin VL (2018). Global, regional, and country-specific lifetime risks of stroke, 1990 and 2016. N. Engl. J. Med..

[2] Wang Y (2015). Ischemic conditioning-induced endogenous brain protection: Applications pre-, per- or post-stroke. Exp. Neurol..

[3] Hoda MN (2014). Remote ischemic perconditioning is effective after embolic stroke in ovariectomized female mice. Transl. Stroke Res..

[4] Lv J (2020). RIPC provides neuroprotection against ischemic stroke by suppressing apoptosis via the mitochondrial pathway. Sci. Rep..

[5] Torres-Querol C, Quintana-Luque M, Arque G, Purroy F (2021). Preclinical evidence of remote ischemic conditioning in ischemic stroke, a metanalysis update. Sci. Rep..

[6] Hougaard KD (2014). Remote ischemic perconditioning as an adjunct therapy to thrombolysis in patients with acute ischemic stroke: A randomized trial. Stroke.

[7] Pico F (2020). Effect of in-hospital remote ischemic perconditioning on brain infarction growth and clinical outcomes in patients with acute ischemic stroke: The RESCUE BRAIN randomized clinical trial. JAMA Neurol..

[8] Bell RM (2022). Remote ischaemic conditioning: Defining critical criteria for success—report from the 11th Hatter Cardiovascular Workshop. Basic Res. Cardiol..

[9] Ren C (2015). Limb remote ischemic per-conditioning in combination with post-conditioning reduces brain damage and promotes neuroglobin expression in the rat brain after ischemic stroke. Restor. Neurol. Neurosci..

[10] Kitagawa K, Saitoh M, Ishizuka K, Shimizu S (2018). Remote limb ischemic conditioning during cerebral ischemia reduces infarct size through enhanced collateral circulation in murine focal cerebral ischemia. J. Stroke Cerebrovasc. Dis..

[11] Nurse P, Hayles J (2011). The cell in an era of systems biology. Cell.

[12] Qiao HJ (2017). Association of lower serum brain-derived neurotrophic factor levels with larger infarct volumes in acute ischemic stroke. J. Neuroimmunol..

[13] Lasek-Bal A (2015). Low concentration of BDNF in the acute phase of ischemic stroke as a factor in poor prognosis in terms of functional status of patients. Med. Sci. Monit..

[14] Zhang HR, Peng JH, Zhu GY, Xu RX (2015). Neuroprotective effects of Bcl-2 overexpression on nerve cells of rats with acute cerebral infarction. Genet. Mol. Res..

[15] Youle RJ, Strasser A (2008). The BCL-2 protein family: Opposing activities that mediate cell death. Nat. Rev. Mol. Cell Biol..

[16] Wang C (2012). Protection by silibinin against experimental ischemic stroke: Up-regulated pAkt, pmTOR, HIF-1α and Bcl-2, down-regulated Bax, NF-κB expression. Neurosci. Lett..

[17] Li J (2018). Limb remote ischemic postconditioning protects integrity of the blood-brain barrier after stroke. Neural. Regen. Res..

[18] Xu CE (2012). Limb remote ischemic postconditioning is effective but also time-course-limited in protecting the brain from I/R injury. Turk. J. Med. Sci..

[19] Ma J (2017). Prevention of the collapse of pial collaterals by remote ischemic perconditioning during acute ischemic stroke. J. Cereb. Blood Flow Metab..

[20] Hirayama Y (2015). Astrocyte-mediated ischemic tolerance. J. Neurosci..

[21] Ploughman M (2009). Brain-derived neurotrophic factor contributes to recovery of skilled reaching after focal ischemia in rats. Stroke.

[22] Geng X (2021). Remote ischemic postconditioning vs physical exercise after stroke: An alternative rehabilitation strategy?. Mol. Neurobiol..

[23] Otsuka S (2016). The neuroprotective effects of preconditioning exercise on brain damage and neurotrophic factors after focal brain ischemia in rats. Behav. Brain Res..

[24] Abe-Dohmae S, Harada N, Yamada K, Tanaka R (1993). BCL-2 Gene is highly expressed during neurogenesis in the central nervous system. Biochem. Biophys. Res. Commun..

[25] Zhang R (2006). Bcl-2 enhances neurogenesis and inhibits apoptosis of newborn neurons in adult rat brain following a transient middle cerebral artery occlusion. Neurobiol. Dis..

[26] Chen J (2000). Suppression of endogenous bcl-2 expression by antisense treatment exacerbates ischemic neuronal death. J. Cereb. Blood Flow Metab..

[27] Hu Q, Luo W, Huang L, Huang R, Chen R (2016). Apoptosis-related microRNA changes in the right atrium induced by remote ischemic perconditioning during valve replacement surgery. Sci. Rep..

[28] Zhang D (2020). RiPerC attenuates cerebral ischemia injury through regulation of miR-98/PIK3IP1/PI3K/AKT signaling pathway. Oxid. Med. Cell Longev..

[29] Uzdensky AB (2019). Apoptosis regulation in the penumbra after ischemic stroke: Expression of pro- and antiapoptotic proteins. Apoptosis.

[30] Krupinski J (2003). Time-course phosphorylation of the mitogen activated protein (MAP) kinase group of signalling proteins and related molecules following middle cerebral artery occlusion (MCAO) in rats. Neuropathol. Appl. Neurobiol..

[31] Ferrer I, Friguls B, Dalfót E, Justicia C, Planas AM (2003). Caspase-dependent and caspase-independent signalling of apoptosis in the penumbra following middle cerebral artery occlusion in the adult rat. Neuropathol. Appl. Neurobiol..

[32] Kim DH, Zhao X (2005). BDNF protects neurons following injury by modulation of caspase activity. Neurocrit. Care.

[33] Rossé T (1998). Bcl-2 prolongs cell survival after Bax-induced release of cytochrome c. Nature.

[34] Salakou S (2007). Increased Bax/Bcl-2 ratio up-regulates caspase-3 and increases apoptosis in the thymus of patients with myasthenia gravis. In Vivo.

[35] Terashi T (2019). Neuroprotective effects of different frequency preconditioning exercise on neuronal apoptosis after focal brain ischemia in rats. Neurol. Res..

[36] Motomura A (2017). Remote ischemic preconditioning protects human neural stem cells from oxidative stress. Apoptosis.

[37] Otsuka S (2021). Effects of detraining on preconditioning exercise-induced neuroprotective potential after ischemic stroke in rats. Brain Struct. Funct..

[38] Otsuka S (2019). Preconditioning exercise reduces brain damage and neuronal apoptosis through enhanced endogenous 14-3-3γ after focal brain ischemia in rats. Brain Struct. Funct..

[39] Sakakima H (2012). Stimulation of functional recovery via the mechanisms of neurorepair by S-nitrosoglutathione and motor exercise in a rat model of transient cerebral ischemia and reperfusion. Restor. Neurol. Neurosci..

[40] Otsuka S (2021). Preconditioning exercise in rats attenuates early brain injury resulting from subarachnoid hemorrhage by reducing oxidative stress, inflammation, and neuronal apoptosis. Mol. Neurobiol..

