# Supplementary material for: Effects of different remote ischemia perconditioning methods on cerebral infarct volume and neurological impairment in rats
Source: Sci Rep. 2023 Feb 7;13:2158. doi: 10.1038/s41598-023-29475-2 (PMC9905538; doi:10.1038/s41598-023-29475-2)
Supplement: Supplementary file 1 — Supplementary Information. [file 41598_2023_29475_MOESM1_ESM.pdf]

# Effects of different remote ischemia perconditioning methods on cerebral infarct volume and neurological impairment in rats

Authors:
Shotaro Otsuka[1,*,†], Yuki Itashiki[2,†], Akira Tani[2], Teruki Matsuoka[2], Seiya Takada[1], Ryoma Matsuzaki[2], Kazuki Nakanishi[2], Kosuke Norimatsu[2], Yuta Tachibe[2], Riho Kitazato[2], Nao Nojima[2], Shogo Kakimoto[2], Kiyoshi Kikuchi[1,3,4], Ikuro Maruyama[1], Harutoshi Sakakima[2,*]

Affiliations:
[1]Department of Systems Biology in Thromboregulation, Kagoshima University Graduate School of Medical and Dental Science, 8-35-1 Sakuragaoka, Kagoshima 890-8520, Japan

[2]Department of Physical Therapy, School of Health Sciences, Faculty of Medicine, Kagoshima University, 8-35-1 Sakuragaoka, Kagoshima 890-8544, Japan

[3]Division of Brain Science, Department of Physiology, Kurume University School of Medicine, 67 Asahi-machi, Kurume, Fukuoka 830-0011, Japan

[4]Department of Neurosurgery, Kurume University School of Medicine, 67 Asahi-machi, Kurume, Fukuoka 830-0011, Japan

[†]These authors contributed equally to this work.

[*]Submitting/corresponding authors' email addresses:
Shotaro Otsuka at k3360022@kadai.jp
Harutoshi Sakakima at sakaki@health.nop.kagoshima-u.ac.jp

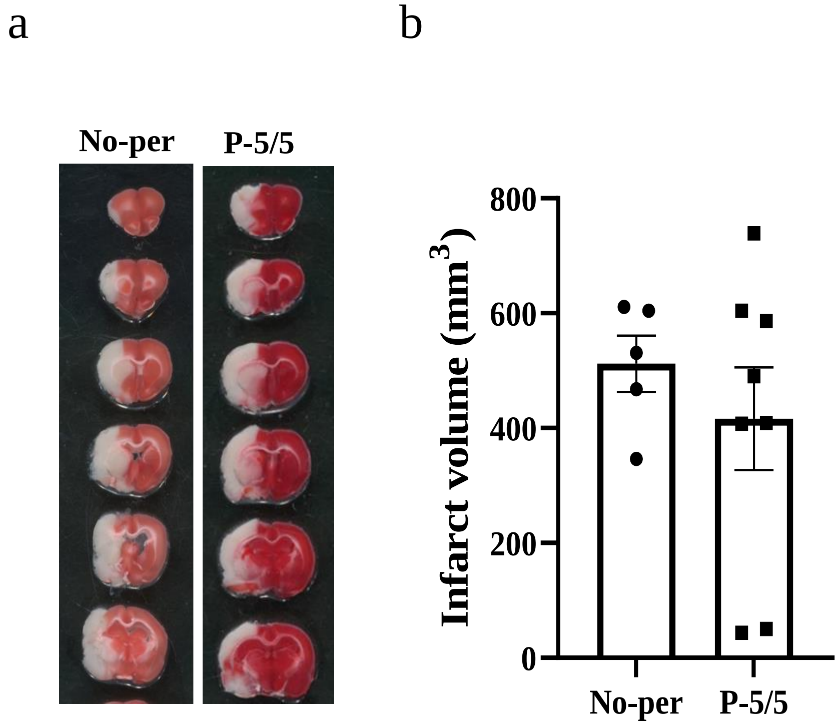


Supplementary Figure 1. Effects of four cycles of 5 minutes of ischemia and 5 minutes of reperfusion RIPerC on cerebral infarct volume.

We evaluated whether four cycles of 5 minutes of ischemia and 5 minutes of reperfusion, as reported in previous studies, reduced cerebral infarct volume. Representative TTC-stained cerebral sections of the No-per group and 4×5-minute ischemia/5-minute reperfusion RIPerC group (P-5/5, n = 8) (a). The infarct volume of the P-5/5 group (416.5 ± 89.3 $mm^3$, $t$ = 0.791, $p$ = 0.445, Student‘s t-test) was not significantly different from that of the No-per group. (b) Data are presented as the mean ± SE.

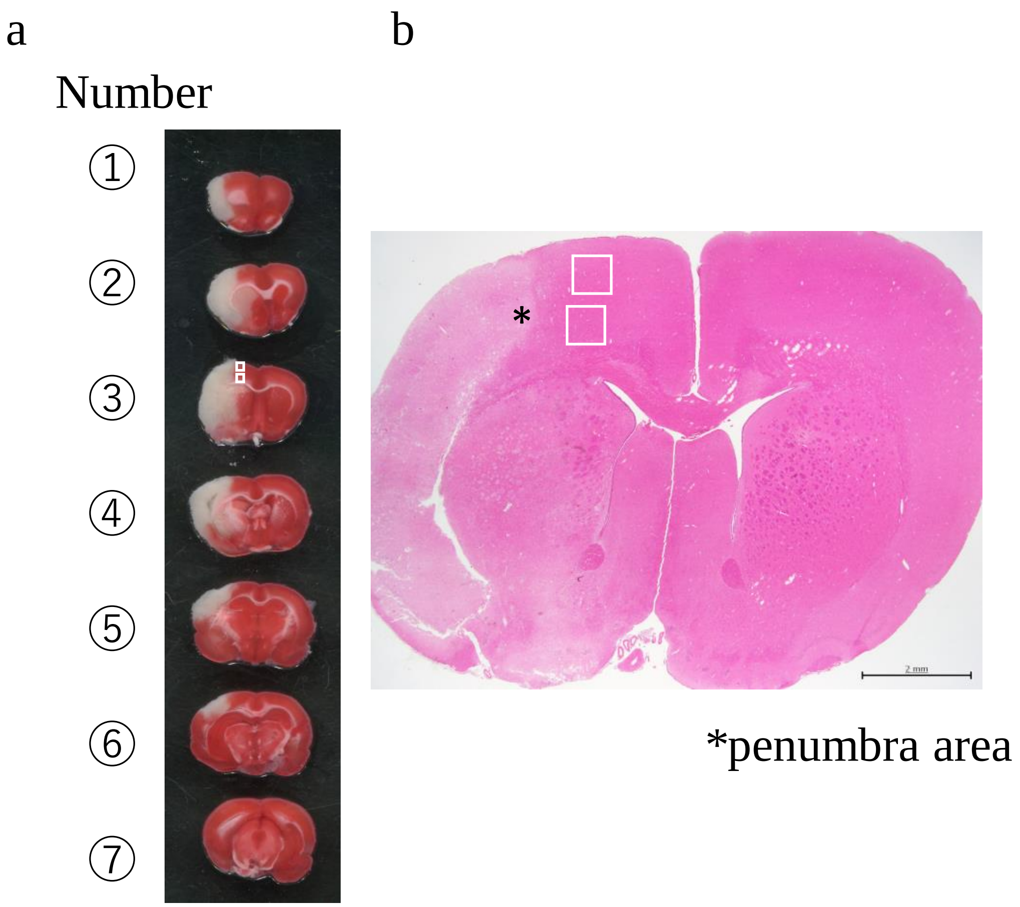


Supplementary Figure 2. Brain sections used for immunohistochemical staining and areas of quantification.

After the brain was removed, seven sections were made at 2 mm intervals using a brain slicer (a). The third of seven tissue sections was used for immunostaining. The two quantified locations are indicated by white squares on the hematoxylin and eosin-stained image (b).

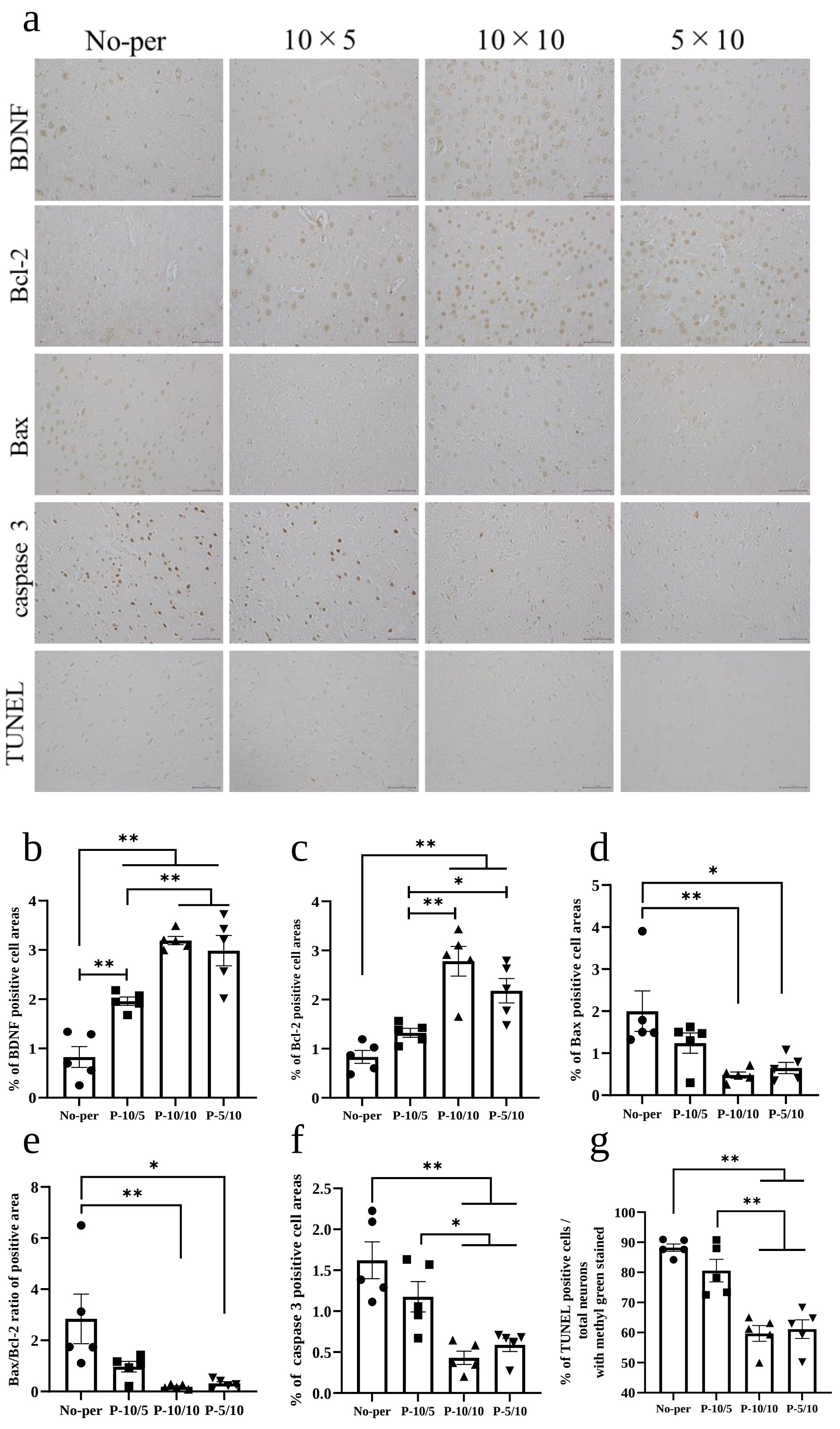


Supplementary Figure 3. Results of second immunohistochemical staining and TUNEL staining experiment.

Photomicrographs of BDNF, Bcl-2, Bax, caspase 3, and TUNEL staining (a); percentage of areas positive for BDNF- (b), Bcl-2- (c), Bax- (d), Bax/Bcl-2 ratio- (e), caspase 3- (f), and TUNEL-positive (g) cells. The same samples as in the first experiment were used. The second experiment yielded similar results to the main text results. Data are presented as the mean ± SE (n = 5). *$p < 0.05$, **$p < 0.01$; scale bar in all panels = 50 μm.

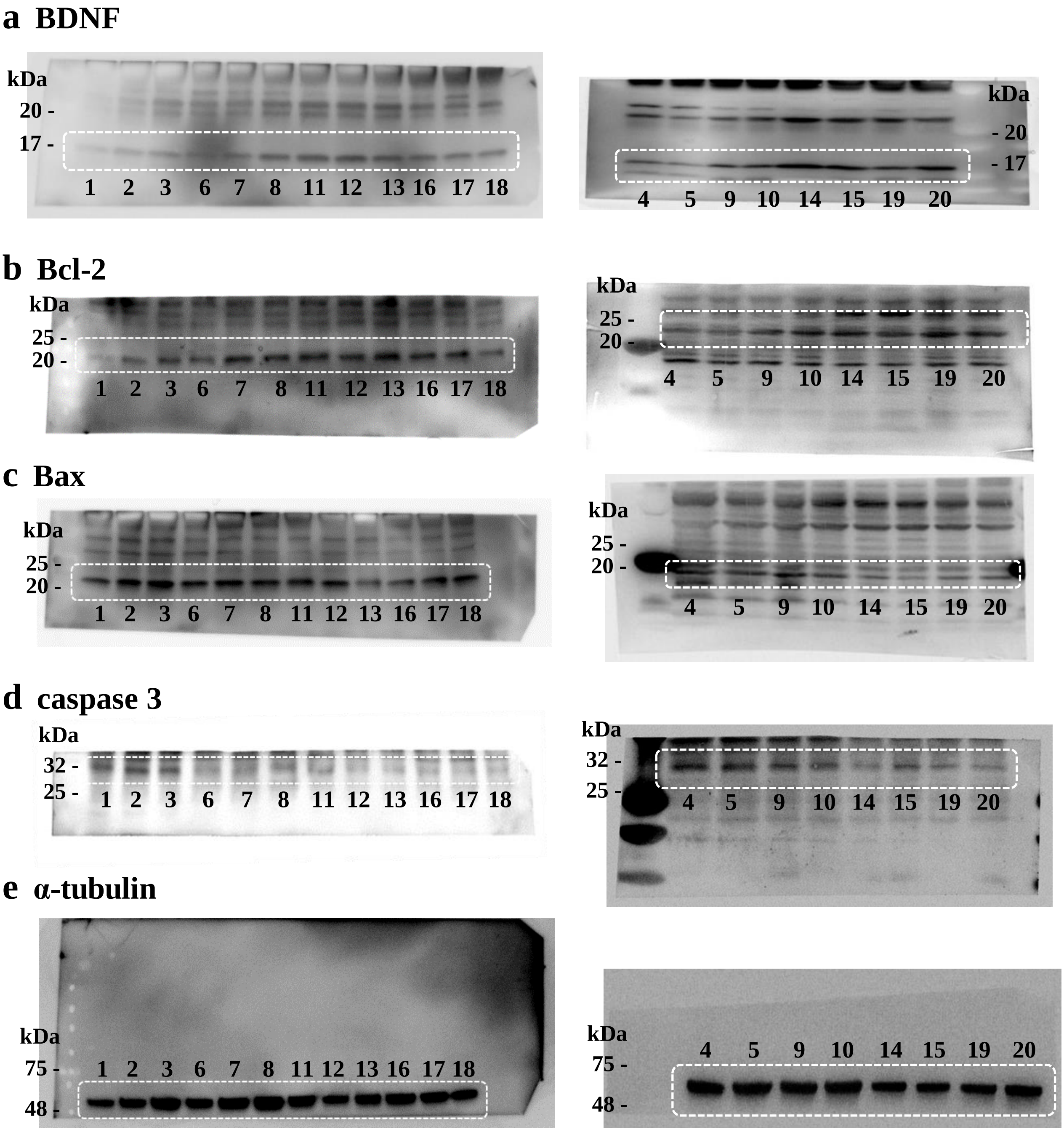


Supplementary Figure 4. Original underlying images for all western blot.
Representative western blots and semi-quantitative analyses of the expression of BDNF (a), Bcl-2 (b), Bax (c), caspase 3 (d), and α-tubulin (e). Protein expression was observed by cutting after transfer from gel to membrane. Each target band is surrounded by a white dotted line. The numbering scheme for each group is as follows; No-per group (1-3), P-10/5 (4-6), P-10/10 (7-9), P-5/10 (10-12).

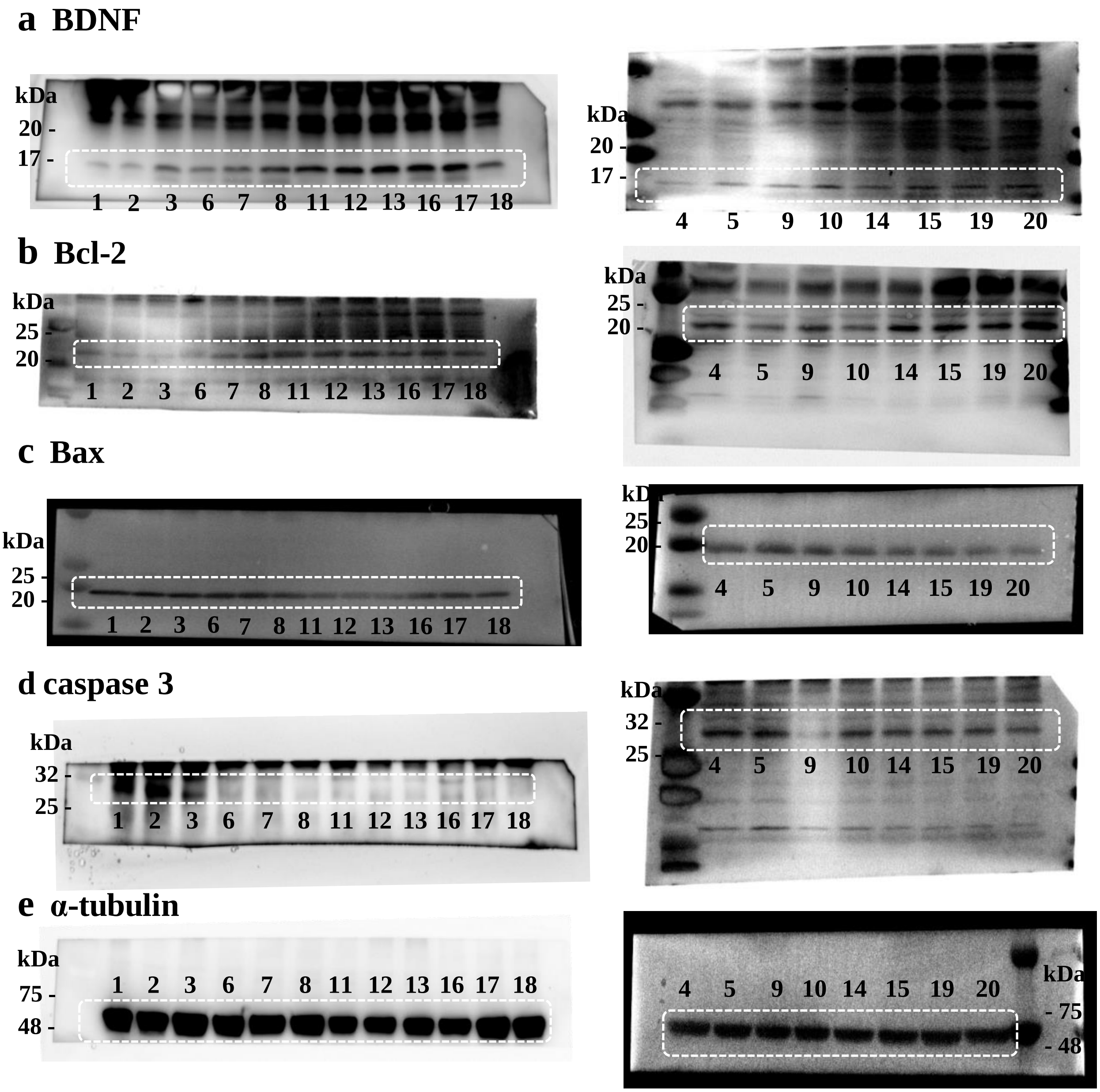


Supplementary Figure 5. Original western blot images from the second experiment. Representative western blots and semi-quantitative analyses of the expression of BDNF (a), Bcl-2 (b), Bax (c), caspase 3 (d), and α-tubulin (e). Protein expression was observed by transferring the protein from the gel to the membrane, cutting the membrane to the appropriate size, and emitting light. Each target band is surrounded by a white dotted line. The same samples as in the first experiment were used. The results followed the same trend as in the first experiment. The numbering scheme for each group is as follows; No-per group (1–5), P-10/5 (6–10), P-10/10 (11–15), P-5/10 (16–20).

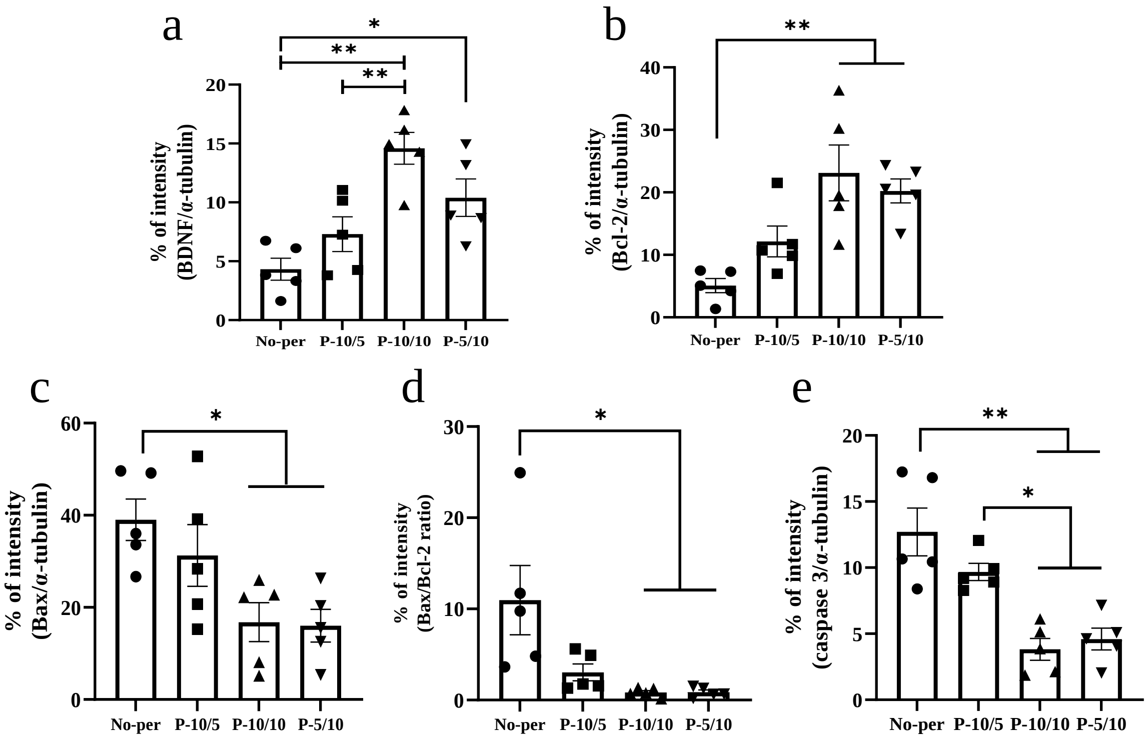


Supplementary Figure 6. Semi-quantitative results of western blots from the second experiment. Representative western blots and semi-quantitative analyses of the expression of BDNF (a), Bcl-2 (b), Bax (c), Bax/Bcl-2 ratio (d), and caspase 3 (e). Protein expression was observed by transferring the protein from the gel to the membrane, cutting the membrane to the appropriate size, and emitting light. Each target band is surrounded by a white dotted line. The results followed the same trend as in the first experiment. Data are presented as the mean ± SE (n = 5). *p < 0.05, **p < 0.01.
